# Development of molecular markers tightly linked to *Pvr4* gene in pepper using next-generation sequencing

**DOI:** 10.1007/s11032-015-0294-5

**Published:** 2015-03-17

**Authors:** Zübeyir Devran, Erdem Kahveci, Ercan Özkaynak, David J. Studholme, Mahmut Tör

**Affiliations:** 1 Department of Plant Protection, Faculty of Agriculture, University of Akdeniz, Antalya, Turkey; 2 M.Y. Genetic Agriculture Technology Laboratory, Antalya, Turkey; 3 Yüksel Seed, Kurşunlu, Madenler Mahallesi, Antalya, Turkey; 4 Biosciences, College of Life and Environmental Sciences, University of Exeter, Stocker Road, Exeter, EX4 4QD UK; 5 National Pollen and Aerobiology Research Unit (NPARU), The University of Worcester, Henwick Grove, Worcester, WR2 6 AJ UK

**Keywords:** Potato virus Y, Pepper, *Pvr4*, Next-generation sequencing, MAS, Synteny

## Abstract

It is imperative to identify highly polymorphic and tightly linked markers of a known trait for molecular marker-assisted selection. *Potyvirus resistance 4* (*Pvr4*) locus in pepper confers resistance to three pathotypes of potato virus Y and to pepper mottle virus. We describe the use of next-generation sequencing technology to generate molecular markers tightly linked to *Pvr4*. Initially, comparative genomics was carried out, and a syntenic region of tomato on chromosome ten was used to generate PCR-based markers and map *Pvr4*. Subsequently, the genomic sequence of pepper was used, and more than 5000 single-nucleotide variants (SNVs) were identified within the interval. In addition, we identified nucleotide binding site–leucine-rich repeat-type disease resistance genes within the interval. Several of these SNVs were converted to molecular markers desirable for large-scale molecular breeding programmes.

**Electronic supplementary material:**

The online version of this article (doi:10.1007/s11032-015-0294-5) contains supplementary material, which is available to authorized users.

## Introduction

Pepper (*Capsicum*) species are among the most important horticultural crops worldwide and belong to the Solanaceae family along with tomato and potato. Cultivated fruits are used as fresh vegetables, spices, colouring agents and for some medical applications (Mathew 2006). Worldwide, approximately 30 million tons per year are produced (FAO 2011). As with other crop plants, pepper is subject to attacks by many pathogens that can significantly reduce yields.

Potato virus Y (PVY) is a member of the group *Potyvirus* and considered to be the most common and important virus in pepper-growing regions (Janzac et al. 2009; Kim et al. 2008; Scholthof et al. 2011). PVY can be transmitted by grafting, sap inoculation and insects such as aphid (Green and Kim 1991; Kanavaki et al. 2006). Isolates of PVY are designated PVY-0, PVY-1, and PVY 1–2 according to their virulence on pepper genotypes (Kyle and Palloix 1997; Caranta et al. 1999).

Since chemical methods have limited success for controlling PVY, resistant varieties would be the most effective means of disease management. Although seven potyvirus resistance genes have been identified in pepper, the *Pvr4* locus has been reported to confer dominant resistance to three pathotypes of PVY (Caranta et al. 1996) and to pepper mottle virus (PepMoV) (Caranta et al. 1999). This dominant gene was derived from the Criollo de Morelos 334 (CM334) variety. Recently, it has been transferred into many pepper varieties using traditional breeding methods where virus tests have been used for selection.

Virus screening assays are useful and utilized commonly in resistance breeding programmes (Ottoman et al. 2009). However, they are laborious, time-consuming and expensive. These difficulties can be overcome by exploiting molecular markers tightly linked to the resistance gene(s). Molecular markers can be used to detect desirable characters at any stage of the plant’s life cycle and reduce time required for phenotypic observation. In the last three decades, several DNA fingerprinting methods have been used for marker development to map relevant genes including restriction fragment length polymorphism (RFLP; Tör et al. 1994), random amplified polymorphic DNA (RAPD; Williams et al. 1990), amplified fragment length polymorphism (AFLP; Rehmany et al. 2000) and cleaved amplified polymorphic sequences (CAPS; Tör et al. 2002). The bulk segregant analysis (BSA) method (Michelmore et al. 1991), which relies on the bulking of around fifteen segregating individual plants to form two pools differing only in the region of interest, has been employed to generate markers closely linked to the gene of interest. Once the markers are identified, a large number of individuals from the segregating populations are tested to confirm the linkage, and subsequently, further markers are developed to use in marker-assisted selection (MAS) programmes.

An AFLP-derived CAPS marker, E41/M49-645, developed previously, is linked to the *Pvr4* locus in pepper (Caranta et al. 1999), close to the marker TG420, which has been placed in 117 cM of chromosome 10 in the integrated map (Lee et al. 2009). In addition, other DNA-based molecular markers have been used for resistance breeding in pepper (Moury et al. 2000; Kim et al. 2008). We attempted to use some of these markers in our pepper-breeding programme. However, we found that the linkage was not close enough to *Pvr4* for a satisfactory MAS programme to assist *Pvr4* introgression into several susceptible backgrounds.

The objective of this study was to develop new molecular markers tightly linked to the disease resistance gene *Pvr4* for molecular breeding in pepper. We employed next-generation sequencing (NGS) technology in combination with the BSA method to generate genomic data from resistant and susceptible lines. Initially, a syntenic region of the tomato genome was used to mine the pepper sequence data that we generated, and hundreds of single-nucleotide variants (SNVs) between pepper and tomato were detected. Several of these SNVs were then converted to MAS-friendly PCR-based markers. Subsequently, the pepper genome sequence became available and was used for fine mapping the locus. The orders of markers and their genetic and physical distance from *Pvr4* were determined using a mapping population.

## Materials and methods

### Virus isolate and biological assay

An isolate of PVY pathotype 1–2 was kindly provided by Eric Verdin (INRA PACA-France) and used throughout this study. The virus was multiplied in susceptible pepper plants (*Capsicum annuum* line Y-CAR) according to previous studies (Boiteux et al. 1996; Dhawan et al. 1996; Echer and Costa 2002). Virus inoculum was prepared by homogenizing infected leaves in 0.01 M phosphate buffer (pH 7.0) containing 0.2 % sodium sulphate. After 600-mesh carborundum was added, cotyledons of test plants at the cotyledon to two true leaf stages were inoculated (Janzac et al. 2009; Kim et al. 2008; Moury et al. 1997, 1998). The plants were then kept in a growth chamber at 22 °C with a 16-h photoperiod. Inoculations were repeated 3–7 days later.

Inoculated plants were evaluated for symptom development 3–4 weeks after inoculation. Plants showing disease symptoms on their uninoculated leaves were rated as susceptible, while those without symptoms were accepted as resistant. After visual evaluation, young leaves were harvested from the plants with and without symptoms on their uninoculated leaves, and DAS-ELISA (Clark and Adams 1977) was performed to determine the presence or absence of the virus.

### Plant lines and generation of mapping population

The susceptible *C. annuum* L. cv. SR-231, a Charleston-type sweet pepper with superior agronomic characters, was crossed with *C. annuum* accession Criollo de Morelos 334, which is resistant to the PVY pathotype 1–2, to generate F_1_ lines. A total of 204 F_2_ seeds were obtained from a single F_1_ plant. Individual plants in the segregating F_2_ lines were then sap-inoculated with the PVY, as described above. Twenty F_2_ resistant lines from these assays were allowed to self-pollinate. Subsequently, twenty-four seedlings from each of these F_3_ lines were sap-inoculated with isolates of PVY to determine their genotypes at the F_2_ stage.

### DNA extraction and sequencing analysis

Genomic DNA was isolated from fresh young leaves by using the Wizard Magnetic Kit (Promega) following the manufacturer’s instructions. The bulked segregant analysis was carried out as previously described (Michelmore et al. 1991). DNA was extracted separately from each individual of the progeny, and DNA from fifteen resistant and fifteen susceptible F_2_ individuals was pooled in equal concentrations to make up the resistant and susceptible bulks, respectively. We generated 1 lane of 100-bp paired-end Illumina HiSeq 2500 sequencing data for each parent (resistant and susceptible) line and bulked (resistant and susceptible) pools, comprising 87.9 M pairs of reads for the susceptible parent, 107.6 M for the resistant parent, 55.2 M for the resistant bulk and 62.3 M for the susceptible bulk. The Illumina reads were first trimmed based on their quality scores using Btrim (Kong 2011) with a cut-off of 25 for average quality scores within a moving window of 5 bp. The minimum acceptable read length was 25 bp (that is, reads that were shorter than 25 bp after trimming were discarded). Other parameters for Btrim were set to default values. *Pvr4* was mapped previously on pepper chromosome 10 (http://solgenomics.net/marker/SGN-M6414/details), and the synteny of the location between tomato and pepper was documented (Wu et al. 2009). We used the interval (59,000,000–61,000,000) from tomato chromosome 10 (RefSeq accession NC_015447) as a reference to align the trimmed sequences using Geneious R7 (created by Biomatters). Once alignments were made, we searched for single-nucleotide variants and other short variants between the parental lines. The alignment results were first converted into BAM format (Li et al. 2009) and visualized using Integrative Genomics Viewer (IGV, James et al. 2011).

Once the sequence of the pepper genome became available (Kim et al. 2014), we used the pepper chromosome 10 sequence version 1.55 [downloaded from the Seoul National University website (http://peppergenome.snu.ac.kr/)]. We extracted the *Pvr4* region and used it as a reference sequence to align our sequences obtained from the Illumina HiSeq 2500. We aligned the tomato and pepper genomic sequences using BLASTN and visualized the alignment results using the Artemis Comparison Tool (Carver et al. 2005). Additional PCR-based markers were generated from the pepper genomic sequences. cDNA databases for CM334 and Zunla-1 were obtained from http://peppergenome.snu.ac.kr/ and http://peppersequence.genomics.cn, respectively.

### Conversion of polymorphic sequences into PCR-based molecular markers

Before SNVs were converted into PCR-based CAPS markers, polymorphic sites were confirmed on both parents and bulks. We then randomly selected candidates to cover the 2 Mb regions, and the SNVs were converted into CAPS marker using dCAPS (http://helix.wustl.edu/dcaps/dcaps.html) (Neff et al. 2002). Each PCR amplification was performed in a total volume of 25 μl containing 20 ng of genomic DNA, forward and reverse primers each at 0.4 μM, 10× PCR buffer, 2 mM MgCl_2_, 0.4 mM dNTPs and 1 U of Taq DNA polymerase (Vivantis). The PCR consisted of a first step at 94 °C for 3 min followed by 35 cycles of 30 s denaturation at 94 °C, 30 s annealing at 50–60 °C (based on *T*_m_ of primers) and 1 min extension at 72 °C. Finally, an extension step was carried out at 72 °C for 5 min. A 10 μl sample of each reaction volume was loaded onto a 1.5 % agarose gel to ascertain whether PCR amplification was successful. The remaining 10–15 μl of PCR were digested with relevant restriction enzymes following manufacturer’s instructions. Digest products of PCR amplicons were separated on a 2 % agarose gel containing TAE buffer at 110 V for 2 h and visualized under UV light after staining with ethidium bromide.

### Confirmation of linkage between established and newly generated markers

Newly generated PCR-based markers were tested first on parents to confirm the polymorphisms and then on a segregating 204 F_2_ population derived from the cross *C. annuum* L. cv. SR-231 × *C. annuum* accession Criollo de Morelos 334. Marker genotyping data and the virus disease phenotyping data were used to identify the *Pvr4* interval. Recombinant lines and the physical map covering the TG420 region were used to narrow the interval for generation of new markers that could be used in the MAS programme. Sequences of PCR-based markers will be provided upon request.

### Accessions

The accession number for Sequenced Read Archive (SRA) is SRX713975.

## Results

### *Pvr4* segregates as a single locus

**Fig. 1:**
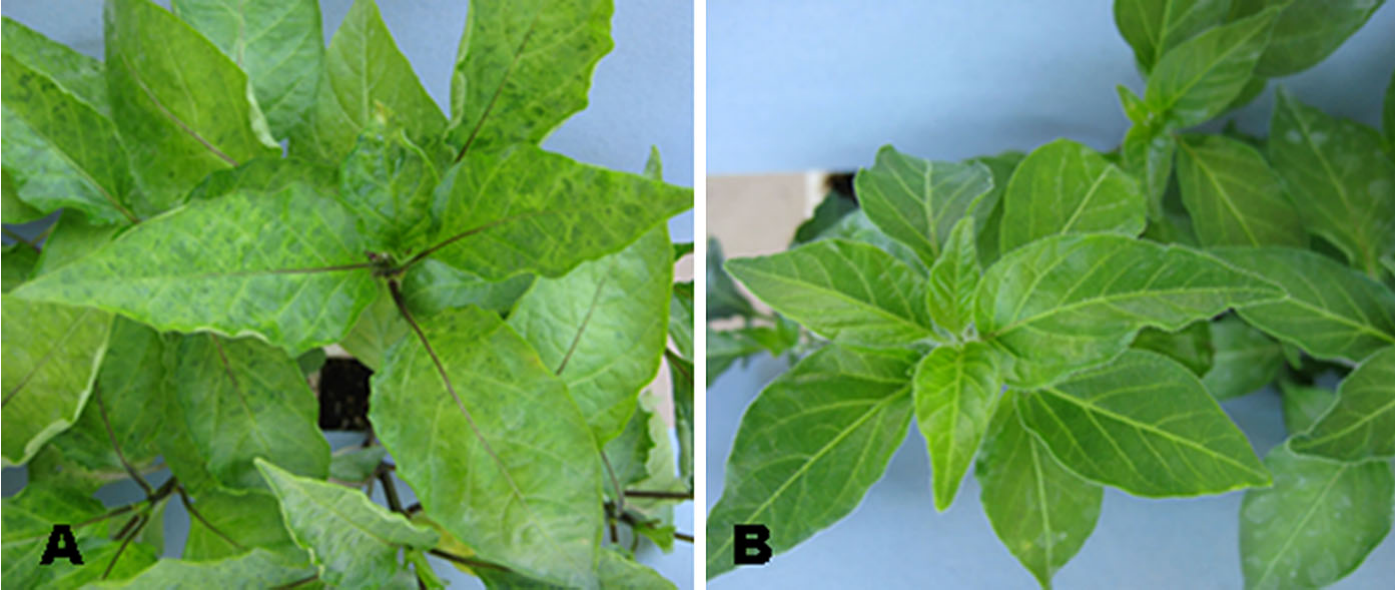
Interaction phenotypes of PVY on pepper cultivars *C. annuum* L. cv. SR-231 (**a**) and Criollo de Morelos 334 (**b**). **a** Susceptible and **b** resistant

*Capsicum annuum* L. cv SR-231 was crossed to *C. annuum* accession Criollo de Morelos 334 (CM334) (Fig. 1). The resulting F_1_ exhibited resistance to PVY 1–2 indicating resistance carried from CM334 was dominant. A population total of 204 segregating F_2_ progeny derived from the F_1_ was inoculated with this virus. The phenotypic observation was confirmed by DAS-ELISA method (Clark and Adams 1977). The observed segregation in this experiment was 150 resistant to 54 susceptible (3:1; *X* ^2^ = 0.05, *P* = 0.05), suggesting that a single gene, *Pvr4*, was the only resistance determinant segregating in this cross.

### Comparative genomics help identify *Pvr4* interval

At the beginning of our study, the pepper genome was not available and the relevant databases (Bombarely et al. 2011) placed *Pvr4* on chromosome 10 towards the telomeric region linked to the marker TG420. In addition, a complete integrated map of pepper was available and a few papers described a genetic interval for *Pvr4* (Caranta et al. 1999; Barchi et al. 2007; Paran et al. 2004; Lee et al. 2009). Since pepper chromosome 10 contains all the markers of the tomato chromosome 10 (Wu et al. 2009) and the tomato genome had recently been sequenced (Tomato Genome Consortium 2012), we focused attention on the region of marker TG420 and used the sequence information from the tomato chromosome 10: 59,000,000–61,000,000 as a reference to align the pepper sequences obtained from parental lines generated with HiSeq 2500 (Illumina). Sequences from the bulked lines were used to confirm the polymorphisms identified.

**Fig. 2:**
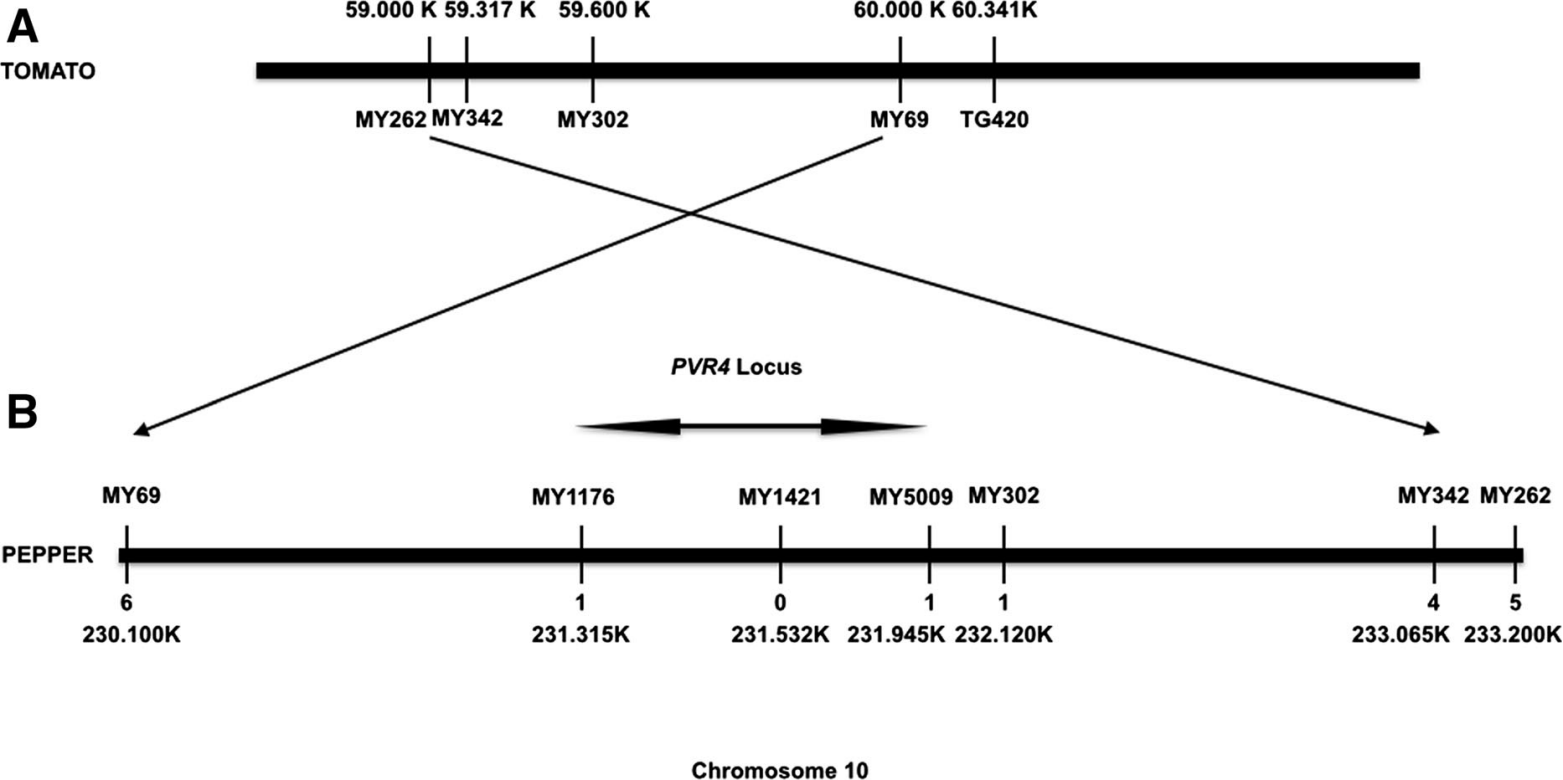
Physical map of *Pvr4* locus in tomato and pepper. **a** *Pvr4* locus in tomato showing the molecular markers around TG420. Markers MY262, MY342, MY302 and MY69 were generated from the pepper sequences aligned to the tomato genome chromosome 10: 59,000,000–61,000,000. **b** *Pvr4* locus in pepper. The region was determined by aligning the *Pvr4* locus in tomato to pepper genome on chromosome 10. Markers MY1176, MY1421 and MY5009 were generated from polymorphic regions of the pepper sequences that were aligned to the pepper genome chromosome 10: 230,000,000–233,200,000. *Numbers* under each marker represents the number of recombinants identified from 387 F_2_ lines

From the resulting alignments against the tomato reference sequence, we identified sites that were polymorphic between resistant and susceptible pepper lines. Some of these polymorphisms consisted of SNVs and were converted into sequence-specific co-dominant PCR-based markers. The CAPS markers MY262 (Tom chr 10: 59,293,491–59,293,668) and MY69 (Tom chr 10:60,111,004–60,111,469) were then used to map *Pvr4* with the segregating F_2_ lines (data for some of the segregating F_2_ lines that are critical for mapping *Pvr4* are given in Supplemental Table 1). A total of 387 F_2_ lines, derived from two individual F_1_ lines, were tested, and there were 5 recombinants for MY262 and 6 for MY69, showing that the markers were linked to *Pvr4.* An interval for the locus was defined in the vicinity of TG420. To reduce the interval, further markers MY342 and MY302 were generated from the polymorphic regions, and mapping was carried out decreasing the interval on the tomato genome to 509 kb (Fig. 2).

### *Pvr4* interval is larger in pepper than that in tomato

**Fig. 3:**
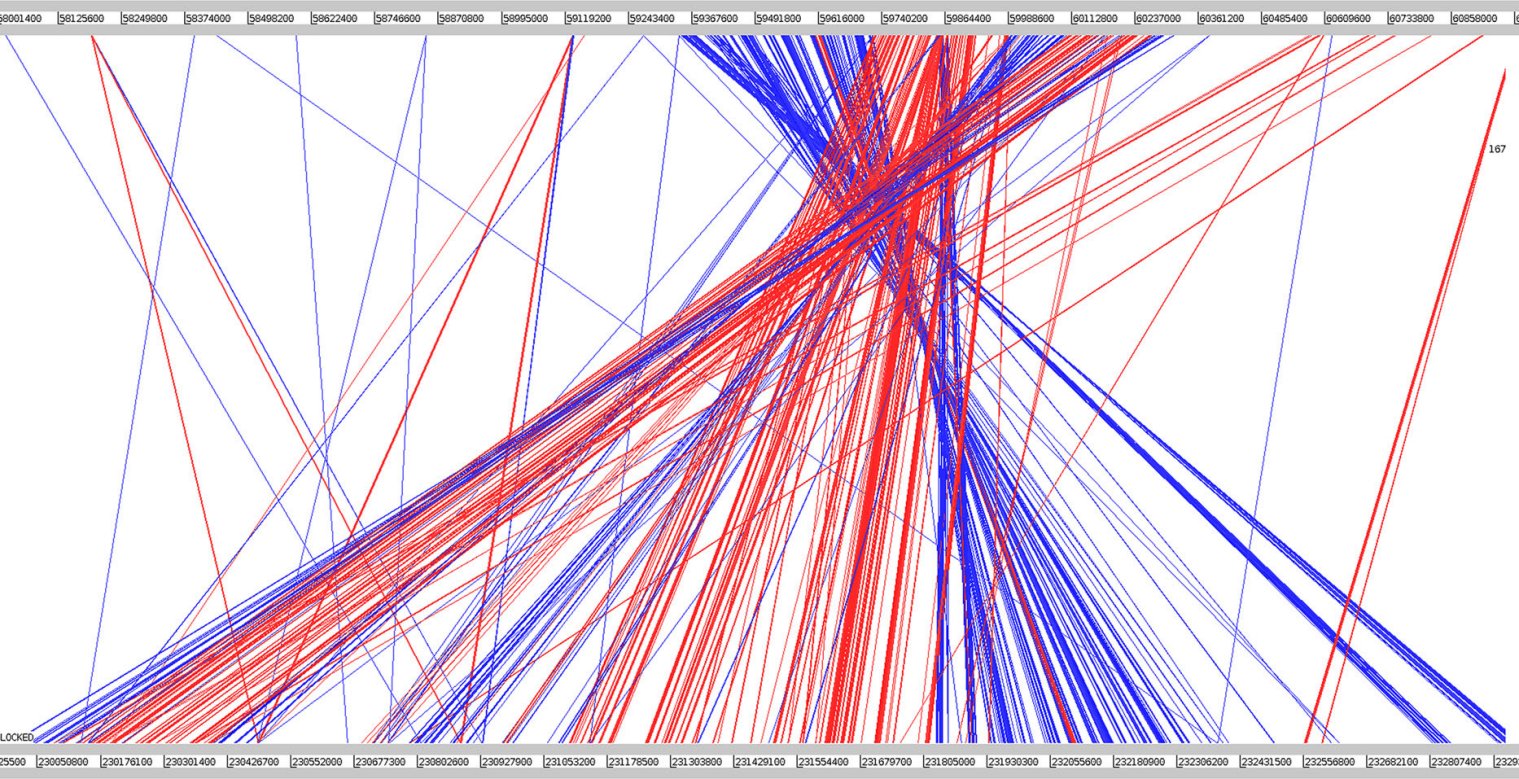
Pairwise sequence alignment of the *Pvr4*-containing region of tomato chromosome 10 versus the pepper chromosome 10. The tomato chromosome sequence version 2.40 (TGR, 2012) was downloaded from the Sol Genomics Network site (ftp://ftp.solgenomics.net/tomato_genome/assembly/build_2.40/). The pepper chromosome sequence version 1.55 (Kim et al. 2014) was downloaded from the Seoul National University website (http://peppergenome.snu.ac.kr/). We aligned the sequences using BLASTN and visualized the alignment results using the Artemis Comparison Tool (Carver et al. 2005). The figure shows only alignments between nucleotides 58,000,000 and 61,000,000 on the tomato chromosome and 230,000,000 and 233,000,000 on the pepper chromosome, and shows only alignments with a BLASTN score of at least 167. Same-strand matches are indicated in *red*, while opposite-strand matches are indicated in *blue*

As the pepper genome became available (Kim et al. 2014), we compared pepper and tomato genomes around the *Pvr4* region using pairwise sequence alignment. There was a high degree of synteny, but this region of chromosome 10 in pepper was inverted and considerably expanded compared to tomato (Fig. 3). Approximately 1 Mb of the tomato chromosome 10 (containing 132 predicted genes) aligns against a 3-Mb region of the pepper chromosome 10 (containing 202 predicted genes). This is broadly consistent with the published observation that the hot pepper genome is fourfold larger than that of tomato and shows an accumulation of Gypsy and Caulimoviridae family elements (Kim et al. 2014). Of the 202 pepper genes in this interval, 92 had a clearly identifiable orthologous candidate in the corresponding tomato interval (Supplemental Table 2). The remaining 110 pepper genes that did not have a clearly identifiable orthologue in the tomato interval were enriched for genes encoding leucine-rich repeats and NB-ARC domains, which are characteristic of many known *R*-genes (25 out of 110 predicted genes); high rates of sequence divergence are well known in *R*-genes and therefore might explain the lack of sequence conservation between pepper and tomato. Also among the genes unique to the pepper interval are several that encode protein domains characteristic of mobile elements, e.g. pepper genes CA10g20550, CA10g21100, CA10g20610, CA10g20600 and CA10g22080, again consistent with the observations of Kim et al. (2014) for the expansion of hot pepper genome compared to that of the tomato.

Further markers were generated from the *Pvr4* region using the now available pepper (version 1.55) chromosome 10 sequences. First, we used the *Pvr4* region between markers MY69 and MY302 (chr10: 230,139,119–232,119,074) as a reference to map short sequences only from parental lines generated by Illumina sequencing; then, we compared the two parents for variations. If the variation frequency was 100 %, these polymorphisms were considered to be suitable to convert to CAPS markers. Using this approach, we identified 5,194 polymorphic sites [insertions, deletions and SNPs] (Supplemental Table 3). Further markers were generated, and *Pvr4* was fine-mapped between MY1176 and MY5009 to an interval of 630 kb with 1 recombinant either side (Fig. 2).

### *Pvr4* interval contains NB-LRR type *R*-genes

Once we had fine-mapped the *Pvr4* gene and identified the interval, we wanted to develop a marker that would be naturally polymorphic for several different pepper varieties. Such a marker could then be easily incorporated into molecular breeding programmes. For this reason, we mapped the Unigene sequences onto the interval using the cDNA data sets generated from *C. annum* cultivars, CM334 and Zunla-1, obtained from http://peppergenome.snu.ac.kr/ and http://peppersequence.genomics.cn, respectively. We then searched the cDNAs aligning within this interval for NBS-LRR- and RLK-type disease resistance genes by using BLASTX. We identified 8 cDNAs in CM334 and 18 in Zunla-1 cultivars that show sequence similarity to NBS-LRR-type *R*-genes (Supplemental Table 4). Since NBS-LRR-type genes can be very polymorphic across different accessions and cultivars, we then generated a new CAPS marker, MY1421, within one of the NBS-LRR-type genes and used it to map *Pvr4* with the F_2_ population. The MY1421 marker co-segregated with *Pvr4* (Fig. 2), indicating its usefulness for MAS during transfer of *Pvr4* into susceptible pepper varieties.

## Discussion

We wanted to generate tightly linked markers for *Pvr4* suitable for molecular breeding programmes. To achieve this, we used a mapping population from a cross between PVY-resistant and PVY-susceptible lines for phenotyping *Pvr4* in the individual progeny plants. Subsequently, we employed NGS technology to sequence the genome of the parental and the bulked lines. We then applied the power of comparative genomics to identify the syntenic region and to reveal polymorphisms between susceptible and resistant lines. Some of the selected polymorphisms were then converted into PCR-based molecular markers, which were then tested on the segregating mapping population to confirm the genetic linkage between the markers and *Pvr4*. Here, we present evidence that *Pvr4* is mapped to an interval of 630 kb with two flanking and one co-segregating markers.

MAS is one of the most widely used applications in breeding programs (Foolad 2007). The process reduces breeding time and allows pyramiding of desirable genes in a superior line. Therefore, development of markers tightly linked to the gene of interest is of high importance for breeders. Previously, DNA-based molecular markers have been developed for resistance breeding in pepper (Mourey et al. 2000; Kim et al. 2008). However, in our previous studies, we have used the published AFLP-derived CAPS marker in our segregating populations and some commercial varieties. Unfortunately, the linkage we observed was not tight enough to carry out MAS programmes (data not shown). In the present study, the power of NGS coupled with comparative genomics led to the development of several markers tightly linked to the target gene *Pvr4*.

Synteny has been described as the preserved order of genes on chromosomes of related species, which results from descent from a common ancestor (Duran et al. 2009). Since tomato and pepper are closely related (both are members of the Solanaceae family) and synteny exists on different parts of the chromosomes, we used comparative genomics to generate markers and map the gene of interest. A 2-Mb genomic sequence from the tomato chromosome 10 around marker TG420 was used, and the short sequences from the parental lines were aligned. SNVs were identified and converted to PCR-based co-dominant markers, and a 509-kb interval for the *Pvr4* was defined. SNPs generated by using NGS technology have been applied to many molecular marker applications including genetic diversity analysis, DNA diagnostics, high-resolution genetic mapping, phylogenetics and selection of desirable characters (Rafalski 2002; Jones et al. 2009). At the beginning of this study, the pepper genome sequence was not available, but the use of NGS enabled us to generate markers rapidly and identify a manageable interval for the gene of interest.

Once the pepper genomic sequence information became available, comparison of the *Pvr4* interval between pepper and tomato genomes revealed that the interval was much bigger in the pepper genome (around 2 Mb) than in tomato. This prompted us to generate further markers by SNV discovery and their conversion to molecular markers. This enabled us to fine-map the *Pvr4* interval in pepper to 630 kb.

Since the markers developed are co-dominant, they can be used to discriminate different alleles in breeding lines and populations. We did not intend to clone the *Pvr4* gene but to identify markers that are tightly linked to it for use in breeding programmes. Molecular markers must be cost-effectively amenable to a large number of samples in order to be used in MAS (Gupta et al. 1999). In addition, molecular markers should co-segregate or be tightly linked to traits of interest, preferably less than 1 cM genetic distance. Thus, the use of flanking markers or intragenic markers greatly increases the reliability of markers to predict phenotype (Ragimekula et al. 2013). In this study, we developed flanking markers with only one recombination event on either side of and less than 1 cM genetic distance away from *Pvr4*. To support this and develop a co-segregating marker, we looked at the possible polymorphic genes within the interval. It is well known that nucleotide binding site–leucine-rich repeat (NB-LRR) proteins confer disease resistance and are the most variable gene family in plants (Guo et al. 2011). Our search for possible NB-LRR cDNAs in the interval revealed eight in the CM334 and 18 in the Zunla-1 cultivars, confirming the usual finding that most NB-LRR genes reside in clusters (Meyers et al. 2003). A marker generated from within one of these genes co-segregated with *Pvr4*.

The number of NB-LRR genes in one cluster can vary between cultivars and species (Guo et al. 2011). This may be the case between the pepper cultivars CM334 and Zunla-1, as well as between tomato and pepper, as indicated by the difference in size of their physical maps of the *Pvr4* locus. In fact, Qin et al. (2014) reported the synteny between tomato and pepper cv Zunla-1 at the gene level. It was clear from their work that out of 18 NB-LRR genes in the interval, only one of them was present in tomato.

In conclusion, the *Pvr4* locus can now be transferred to superior pepper lines via marker-assisted backcross selection. Since genetic variation is high in pepper genome, the markers developed in this study could easily be tested for efficiency in breeding lines with different genetic backgrounds. Our findings contribute to the improvement and generation of new hybrid pepper lines.

## Electronic supplementary material

Below is the link to the electronic supplementary material.

Supplementary material 1 (XLSX 11 kb)

Supplementary material 2 (XLSX 222 kb)

Supplementary material 3 (XLSX 9 kb)

Supplementary material 4 (XLSX 22 kb)
